# Supplementary figures and images for: Polymer coil–globule phase transition is a universal folding principle of Drosophila epigenetic domains
Source: Epigenetics Chromatin. 2019 May 13;12:28. doi: 10.1186/s13072-019-0269-6 (PMC6515630; doi:10.1186/s13072-019-0269-6)

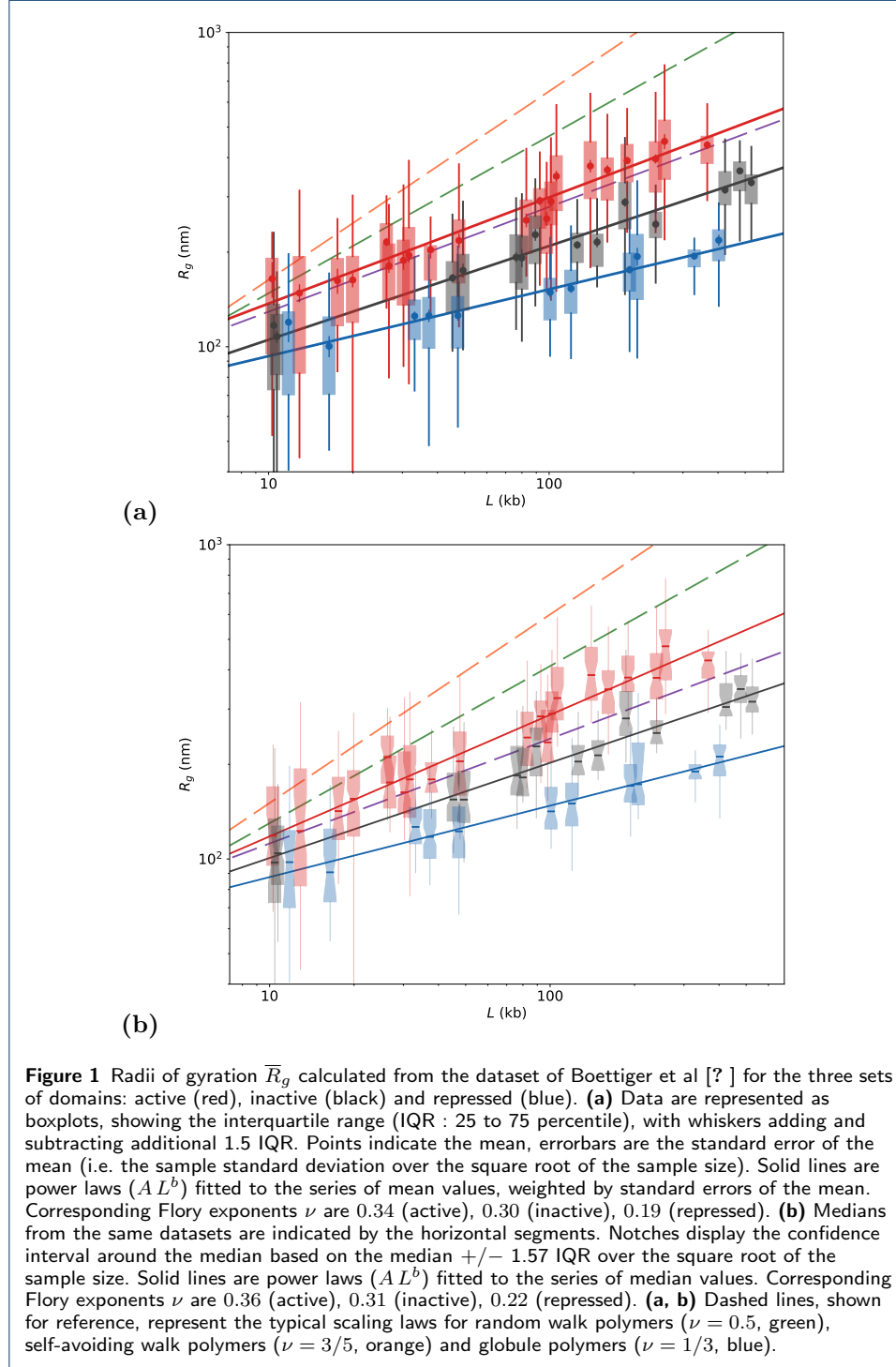

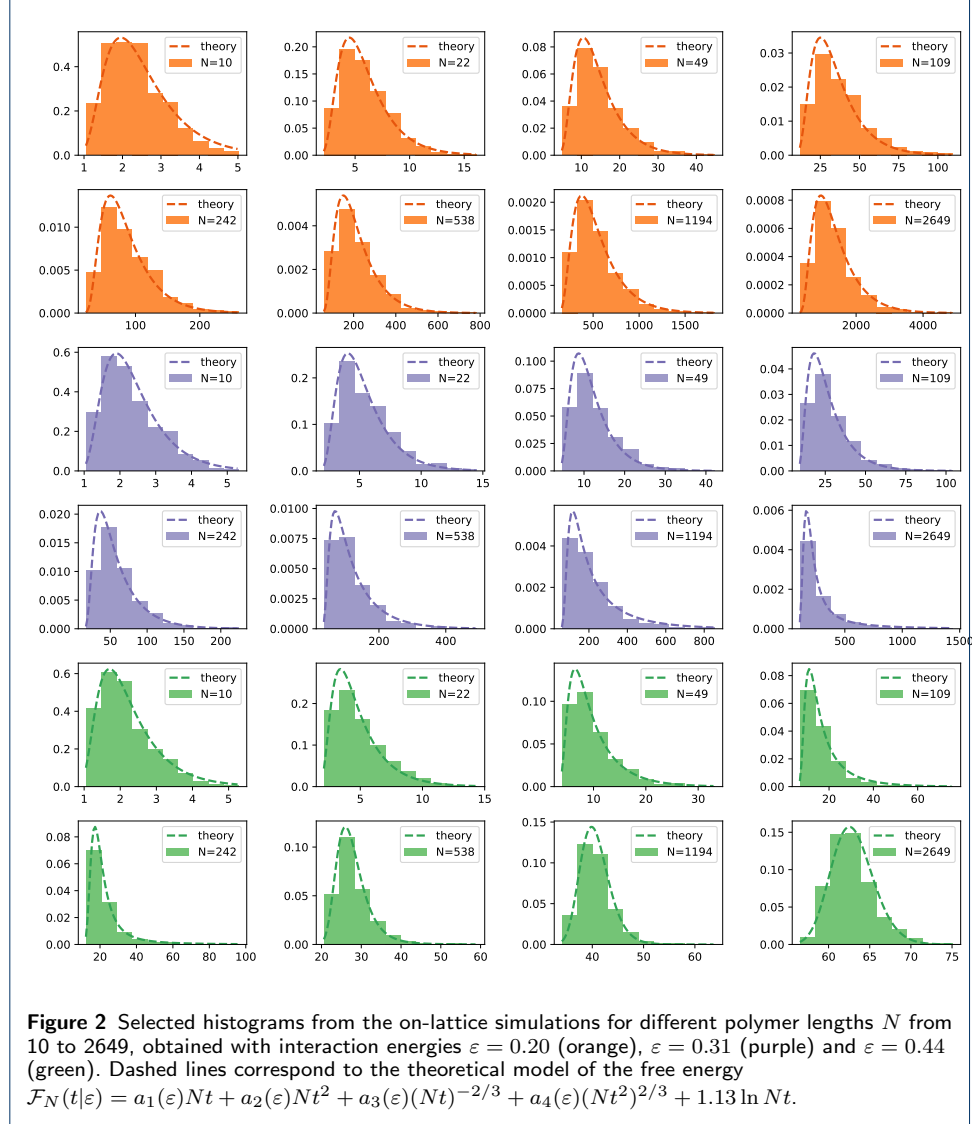

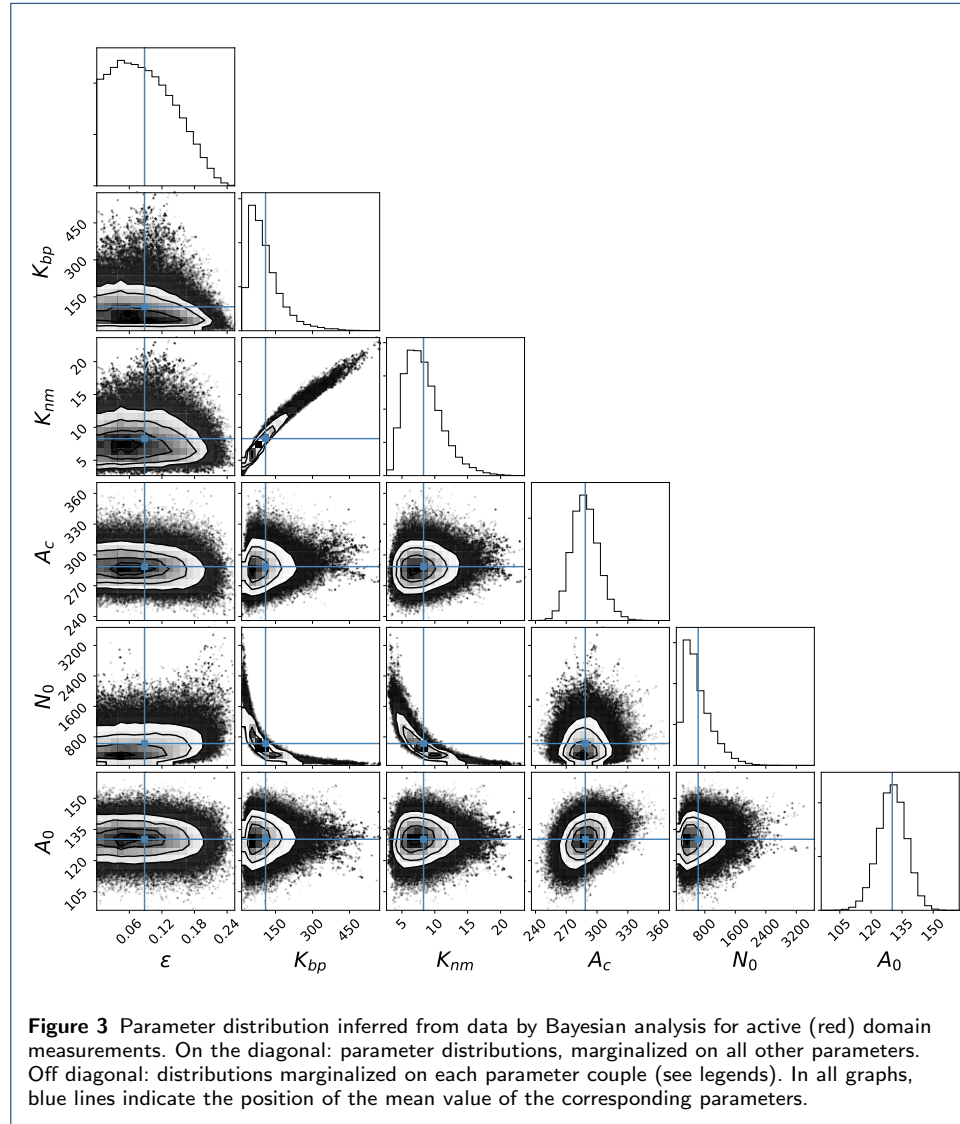

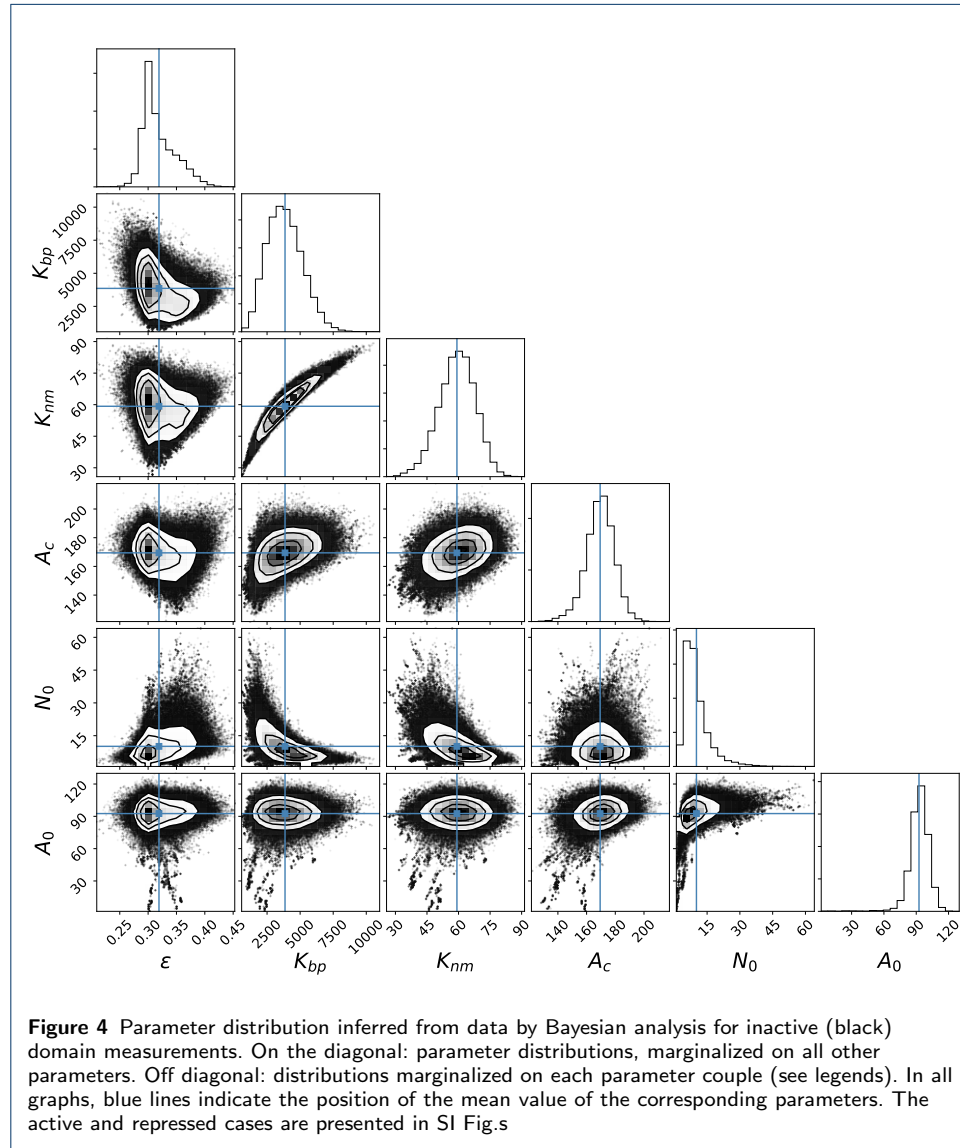

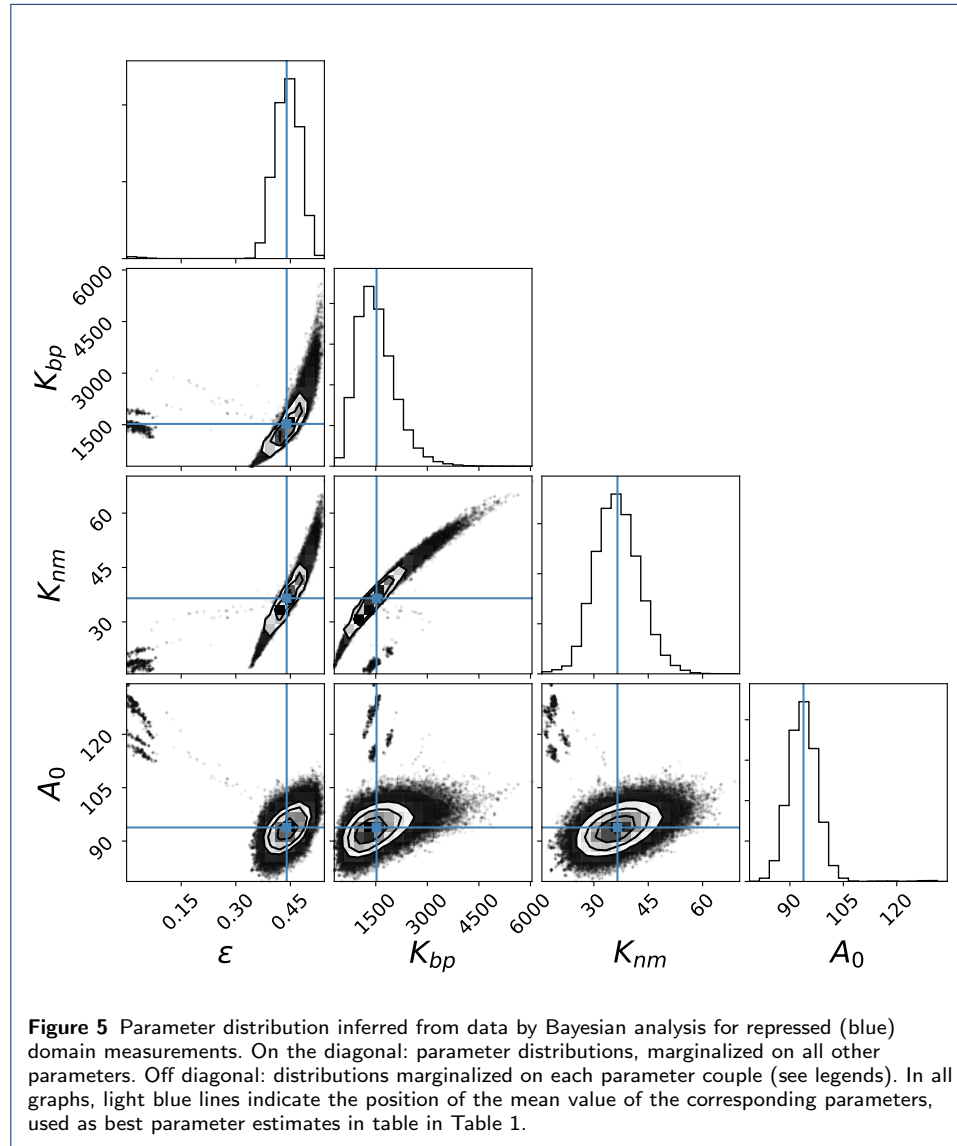

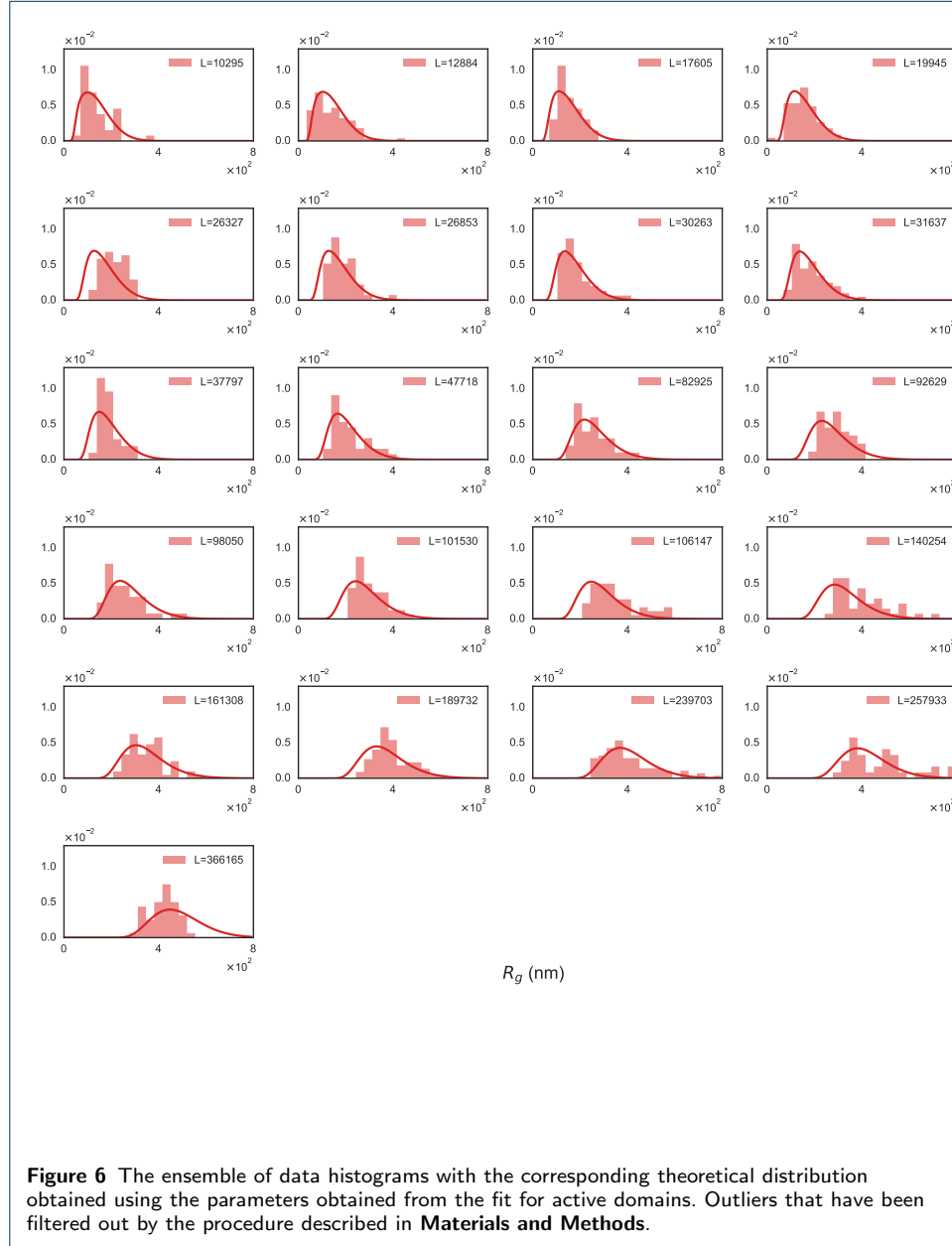

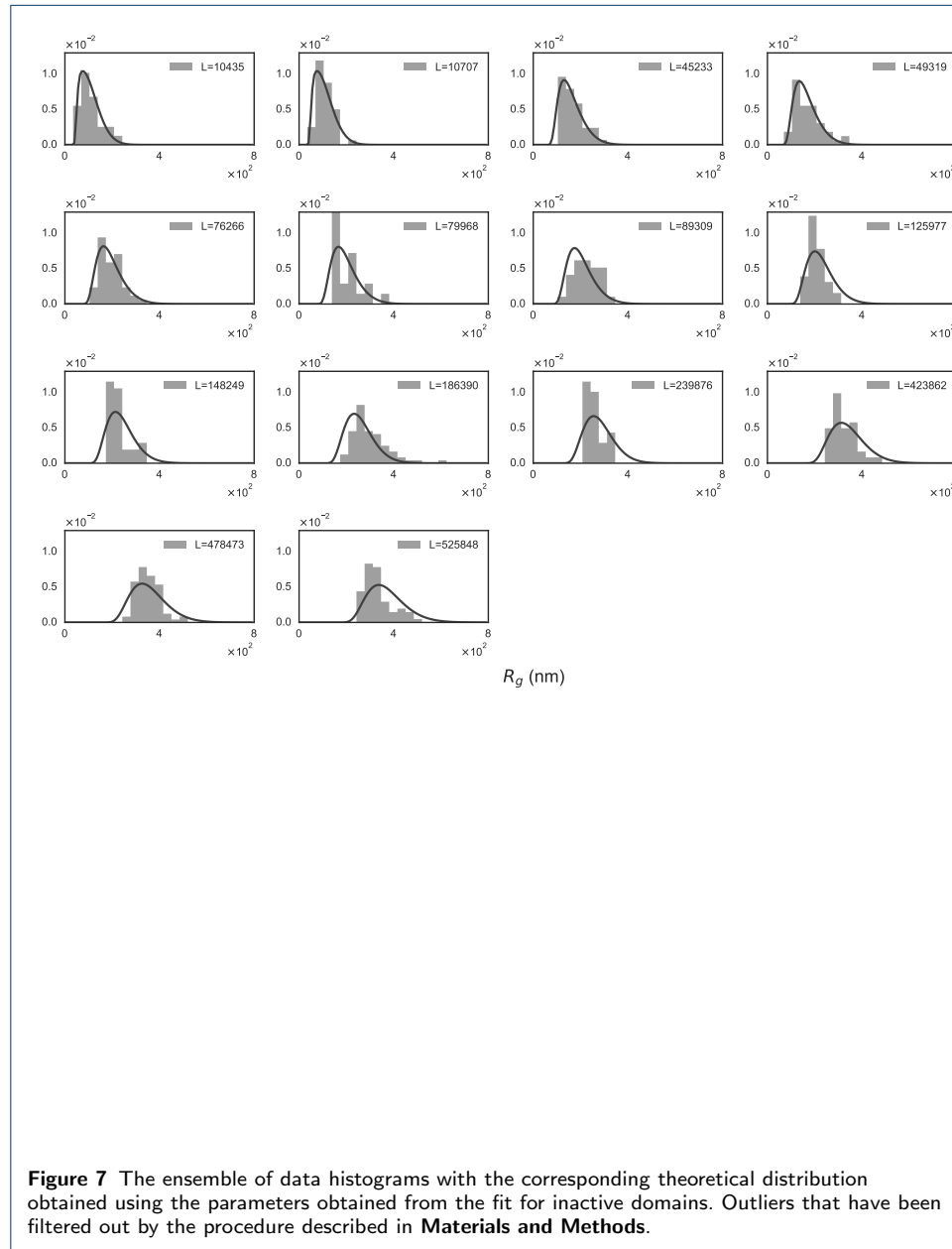

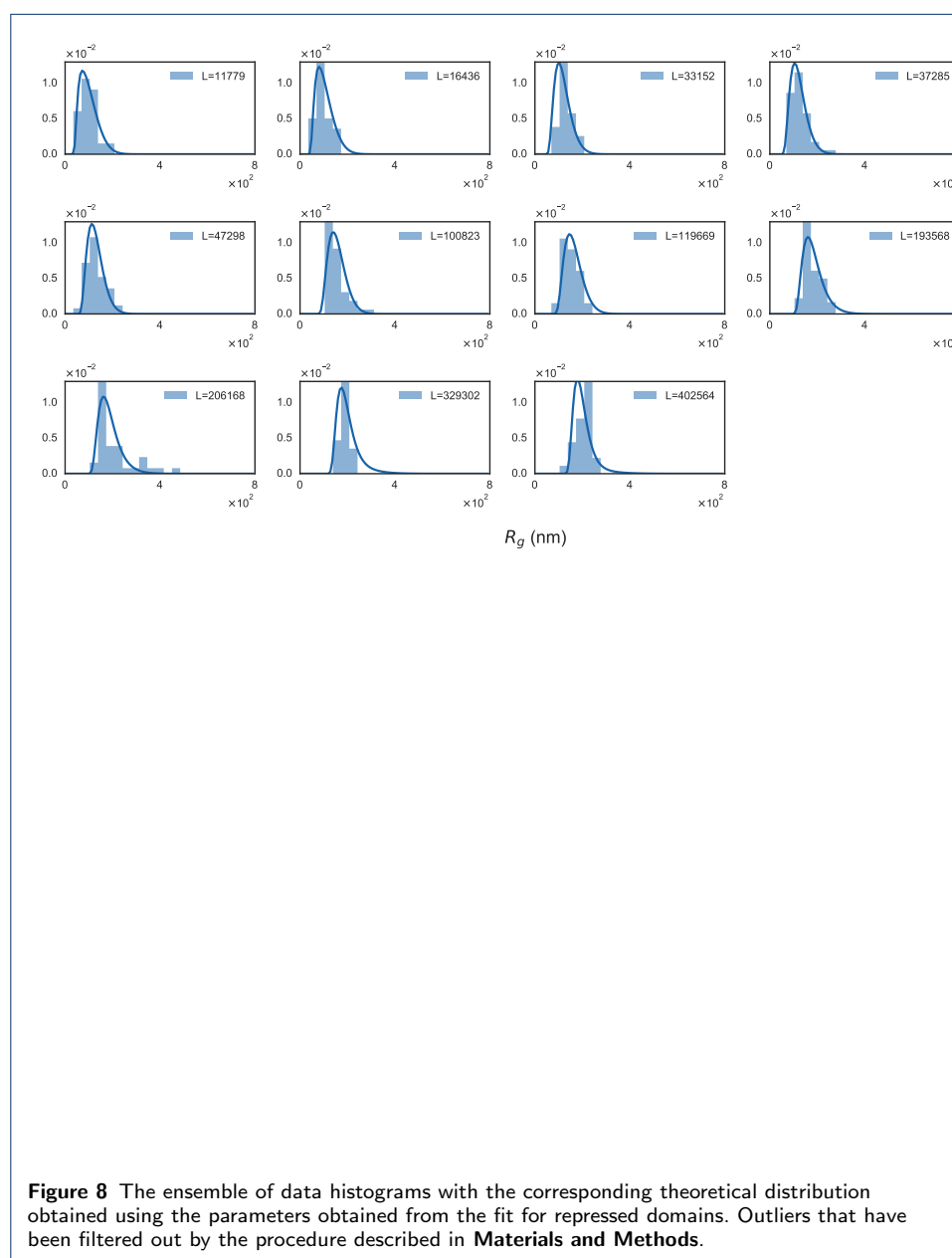

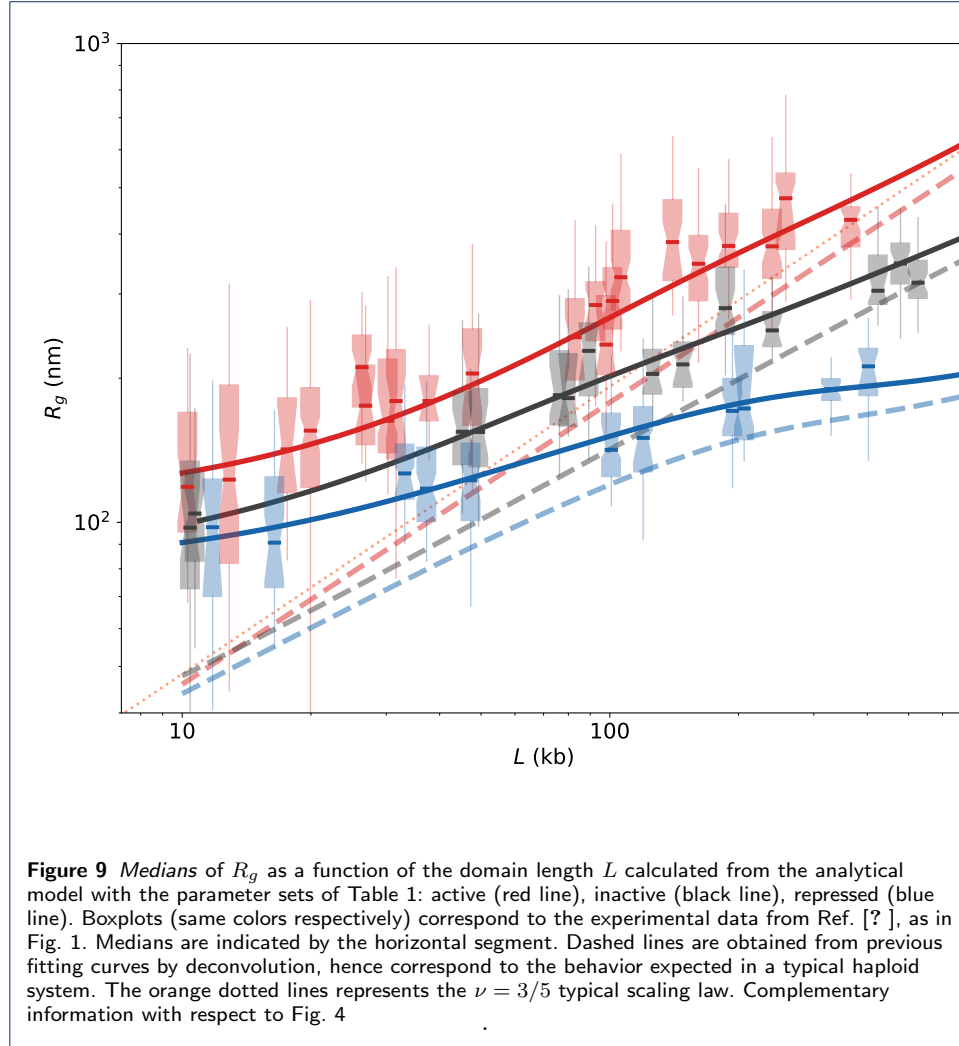

Supplement: Supplementary file 1 — Additional file 1: Figure S1. Mean and median radii of gyration from the dataset of Boettiger et al. [2] with corresponding boxplots. Figure S2. Selected histograms from the on-lattice simulations with the corresponding theoretical distributions. Figures S3, S4, S5. Parameter distributions inferred from data by Bayesian analysis for active, inactive and repressed domains, respectively. Figures S6, S7, S8. Data histograms with the corresponding theoretical distributions obtained using the fitted parameters for active, inactive and repressed domains, respectively. Figure S9. Gyration radius experimental medians and boxplots with the corresponding fitting theoretical curves. [file 13072_2019_269_MOESM1_ESM.pdf]
